# Examining the Incidence and Predictors of Low Resilience and Potential PTSD Among Residents in Two Canadian Provinces Amidst the 2023 Wildfires

**DOI:** 10.1192/j.eurpsy.2024.711

**Published:** 2024-08-27

**Authors:** M. K. Adu, R. Shalaby, B. Agyapong, R. D. L. Dias, V. I. Agyapong

**Affiliations:** ^1^Department of Psychiatry, Dalhousie University, Halifax, NS; ^2^Department of Psychiatry, University of Alberta, Edmonton, AB, Canada

## Abstract

**Introduction:**

The recent wildfires in Canada provide a clear illustration of the significant and lasting damage they inflict on the well-being of individuals and communities. Evaluating the occurrence and factors associated with post-traumatic stress disorder (PTSD) and low resilience is valuable for policymakers in public health.

**Objectives:**

The study aimed to assess the prevalence and predictors of low resilience and likely PTSD among subscribers of Text4Hope, an e-mental health program that delivered daily supportive messages to residents of Nova Scotia (NS) and Alberta (AB) during the recent wildfires.

**Methods:**

Data collection was through a self-administered online survey completed by residents of the affected regions of NS and AB from May 14 to June 23, 2023. Data were analyzed using Statistical Package for the Social Sciences.

**Results:**

Out of 298 respondents, the prevalence of low resilience and likely PTSD in our sample were 52.0% and 39.3% respectively. Unemployed respondents were about 3 times more likely to experience both low resilience and PTSD symptoms compared to those employed. Respondents with a history of mental health diagnosis were about 4 times more likely to experience likely PTSD compared to those with no history of mental health diagnosis.

**Conclusions:**

This research demonstrated that the likelihood of PTSD was predicted by both unemployment and a history of mental health diagnosis, with unemployment also being linked to low resilience during the wildfire. These results provide valuable insights for designing clinical interventions and developing psychosocial support programs tailored for vulnerable populations.

**Disclosure of Interest:**

None Declared

